# Adverse childhood experiences and early life inflammation in the Avon longitudinal study of parents and children

**DOI:** 10.1016/j.psyneuen.2020.104914

**Published:** 2020-12

**Authors:** Rebecca E Lacey, Mel Bartley, Michelle Kelly-Irving, Leonardo Bevilacqua, Eleonora Iob, Yvonne Kelly, Laura D Howe

**Affiliations:** a Research Department of Epidemiology and Public Health, University College London, 1-19 Torrington Place, London WC1E 6BT, United Kingdom; b INSERM, Université Toulouse III Paul-Sabatier, France; c Department of Behavioural Science and Health, University College London, 1-19 Torrington Place, London WC1E 6BT, United Kingdom; d Medical Research Council Integrative Epidemiology Unit at the University of Bristol, Population Health Sciences, Bristol Medical School, University of Bristol, United Kingdom

**Keywords:** Adverse childhood experiences, Adversities, ALSPAC, C-reactive protein, Interleukin-6, Inflammation

## Abstract

**Background:**

Adverse childhood experiences (ACEs) have been associated with poorer health across the life course. Previous studies have used cumulative risk scores (ACE scores) or individual ACEs but these two approaches have important shortcomings. ACE scores assume that each adversity is equally important for the outcome of interest and the single adversity approach assumes that ACEs do not co-occur. Latent class analysis (LCA) is an alternative approach to operationalising ACEs data, identifying groups of people co-reporting similar ACEs. Here we apply these three approaches for ACEs operationalisation with inflammation in childhood with the aim of identifying particular ACEs or ACE combinations that are particularly associated with higher inflammation in early life.

**Methods:**

Using data from the Avon Longitudinal Study of Parents and Children (ALSPAC) we compare ACE scores, single adversities and LCA-derived ACE clusters in their relationships with Interleukin-6 at age 9 (n = 4935) and C-Reactive Protein (CRP) at age 9 (n = 4887). ACEs included were parental separation/divorce, parental alcohol problems, parental mental health problems, parental offending, inter-parental violence, parental drug misuse, and physical, emotional and sexual abuse.

**Results:**

Two thirds of the sample reported at least one ACE. Mother’s mental health problems was the most frequently reported ACE (32.3 %). LCA identified four ACE classes – ‘Low ACEs’ (81.1 %), ‘Maternal mental health problems’ (10.3 %), ‘Maternal mental health problems and physical abuse’ (6.3 %) and ‘Parental conflict, mental health problems and emotional abuse’ (2.4 %). Parental separation/divorce was associated with higher IL-6. Parental alcohol problems, paternal mental health problems, parental convictions and emotional abuse were associated with lower levels of IL-6. Associations for paternal mental health problems and emotional abuse were only observed for boys. ACE score and LCA-derived ACE classes were not associated with differences in IL-6. Girls in the ‘Maternal mental health problems’ cluster had lower CRP levels.

**Conclusions:**

Specific adversities and adversity combinations are important for differences in childhood inflammation. Some associations were only observed for girls or boys.

## Highlights

• There is a reliance on ACE scores and single adversity approaches in ACEs research.
• We compare ACE scores, individual ACEs and latent class analysis (LCA) for early life inflammation.
• LCA identified four clusters. ‘Maternal mental health problems’ was associated with lower CRP for girls.
• IL-6 was higher for parental divorce and lower for emotional abuse, paternal mental health problems, parental offending and alcohol misuse.
• Associations between paternal mental health problems and emotional abuse with lower IL-6 were only seen for boys.

## 1 Introduction

The importance of adverse childhood experiences (ACEs) for lifelong health have been well documented. The research thus far has largely shown that people experiencing ACEs are at higher risk of numerous adverse child and adult health outcomes e.g.(Hughes et al., 2017), on average. Chronic inflammation is thought to be one of the key mechanisms through which ACEs influence long-term disease outcomes, such as cardiovascular disease and depression (Baldwin and Danese, 2019; Danese and Baldwin, 2017). Indeed, childhood adversities have been associated with altered inflammatory responses (Chen and Lacey, 2018; Danese et al., 2011, 2008, 2007; Slopen et al., 2013; Taylor et al., 2006). Specific ACEs linked to higher levels of C-Reactive Protein (CRP) or Interleukin(IL)-6 have included child maltreatment (Danese et al., 2008), parental mental health disorders (O’Connor et al., 2019) and parental separation/divorce (Lacey et al., 2013). There have been many studies investigating associations between ACEs and inflammatory markers in adulthood, but less research has explored associations between ACEs and inflammatory markers in childhood enabling us to assess whether differences in inflammatory markers are present in childhood (Kuhlman et al., 2020).

### 1.1 Operationalising ACEs

#### 1.1.1 ACE scores

Thus far, the relationship between ACEs and inflammation has largely been tested using ACE scores, whereby the number of ACEs are summed. Results of these studies typically show a graded association between the ACE score and inflammation, with more ACEs being associated with higher levels of inflammation (Baldwin et al., 2018; Chen and Lacey, 2018; Iob et al., 2019; Rasmussen et al., 2019; Slopen et al., 2015). However, the limitations of ACE scores for research and practice are becoming increasingly recognised (Hartas, 2019; Kelly-Irving and Delpierre, 2019; Lacey and Minnis, 2020). Each ACE in the ACE score is assumed to be equally important for the outcome of interest and the mechanisms linking them are assumed to be the same (McLaughlin et al., 2014). Consequently, the specific patterning or combinations of ACEs are ignored (Lacey and Minnis, 2020). There is therefore a need to explore the relationships between different ACEs or ACE clusters with health. This would help us identify which ACEs or combination of ACEs put people most a risk of poor health.

#### 1.1.2 Individual ACEs

Evidence is emerging showing that the specific types of adversity experienced might show distinct physiological pathways to later health. For example, in the 1958 British birth cohort physical abuse and parental offending were particularly strongly associated with higher levels of CRP, fibrinogen and von Willebrand Factor (vWF) in mid-life, however no associations were observed for parental substance misuse, parental mental illness, sexual abuse, and parental death (R. E. Lacey et al., 2020b). Also in relation to adolescent inflammation, parental absence and sexual abuse were associated with higher CRP at age 16 but physical abuse and verbal abuse were not (Jonker et al., 2017)..

There is evidence that the Hypothalamic-Pituitary-Adrenal (HPA) axis and immune systems respond and react differently to different stressors (Kuhlman et al., 2017, 2015; Sheridan and McLaughlin, 2014). Sheridan and McLaughlin (2014) proposed a theoretical dimensional ACEs framework in which ACEs involving experiences of threat (e.g. physical abuse and interparental conflict) - as opposed to ACEs involving experiences of deprivation (e.g. institutionalisation) – are linked to neural development through different mechanisms. Empirical validation of this hypothesis has begun, with the authors finding that interpersonal violence (treated as a threat adversity) was associated with blunted HPA-axis and sympathetic nervous system activity, but poverty (treated as a deprivation adversity) was not (Busso et al., 2016). Similarly, exposure to threat-related adversities was related to accelerated DNA methylation age and advanced pubertal stage, but deprivation-related adversities were not (Sumner et al., 2018).

#### 1.1.3 Person-centred approaches

Whilst focusing on individual ACEs has merit, it ignores the potential clustering of ACEs (Lacey and Minnis, 2020). In the Kaiser Permanente ACE study, between 81 and 98 % of participants reporting one ACE reported at least one other (Dong et al., 2004). Person-centred approaches, e.g. latent class analysis (LCA), are increasingly being used to identify groups or clusters of individuals reporting similar co-occurring ACEs (Lacey and Minnis, 2020). This method allows researchers to explore the clustering of ACEs in population samples and differential associations with key predictors or outcomes (e.g. inflammation). For instance, Lacey et al. (2020) separated out prospective and retrospectively-reported ACEs data in the 1958 British birth cohort, finding three LCA-derived ACE clusters in the prospective data and four in the retrospectively reported ACEs data. In the prospective data, ‘Parental loss’ and ‘Household dysfunction’ were both associated with higher inflammation in mid-life compared to the ‘Low ACEs’ class. Also, in the retrospective data, those in the ‘Maltreatment and conflict’ and ‘Polyadversity’ (experiencing many adversities) classes had higher levels of inflammation in mid-life compared to the ‘Low ACEs’ group, but ‘Parental mental health and substance misuse’ were not. Thus far, LCA has not been applied to studies of ACEs and early life inflammation to enable us to explore whether different ACE clusters are associated with inflammation earlier in the life course

#### 1.1.4 Comparing ACE operationalisations

Few studies have compared at least two of these ACE operationalisations with respect to health outcomes. Merians et al. (2019) compared ACE scores and LCA derived ACE clusters in a study of US college students. Both approaches yielded similar findings when investigating associations with mental and physical health outcomes. However one study using the US National Longitudinal Study of Adolescent to Adult Health found that latent ACE variables derived by factor analysis had greater explanatory power for predicting outcomes compared to an ACE score (Brumley et al., 2019). With respect to inflammatory markers, Iob et al. (2019) used the English Longitudinal Study of Ageing (ELSA) to compare associations between ACE scores and ACE dimensions extracted via factor analysis. They found that ACE scores were related to CRP in later life and that similar associations were seen for different ACE dimensions, suggesting that the number of ACEs reported was most important for later-life inflammation.

Only one previous study, to the authors’ knowledge, has compared these three methods of operationalising ACEs - ACE scores, individual ACEs and LCA-derived ACE clusters - in relation to inflammation. Lacey et al. (2020) explored associations between these three methods of operationalising ACEs with inflammatory markers in mid-life in the 1958 British birth cohort. Many individual ACEs were strongly related to inflammation, particularly parental offending and physical abuse. In the 1958 British birth cohort the LCA on the prospective ACEs data reflected the associations shown with individual ACEs and therefore did not add much to the understanding of associations with inflammation in mid-life. In the retrospectively-reported ACEs data, where there were stronger correlations between ACEs, the LCA clusters representing ‘Polyadversity’ and ‘Maltreatment and conflict’ showed the strongest associations with inflammatory markers, akin to an ACE score of 4 + . But the strongest associations were seen between physical abuse, family conflict and psychological abuse, when considered individually. As yet, no one has investigated associations between these three methods of ACE operationalisations and inflammatory markers earlier in life to assess whether differences in inflammatory responses emerge earlier.

#### 1.1.5 Gender differences

Previous research has shown that there are gender differences in who experiences different types of ACEs. For instance, girls are more likely to report sexual abuse and boys more likely to report physical abuse or witnessing abuse (Hanson et al., 2008; Lacey et al., 2020a; Leban and Gibson, 2019). Whilst research on the Avon Longitudinal Study of Parents and Children (ALSPAC) has shown that the clustering of ACEs does not differ for girls and boys (Lacey et al., 2020a), the relationships between individual ACEs, ACE scores and LCA-derived ACE clusters with early life inflammation may differ by gender. Research has shown that an ACE score of 4+ was associated with DNA methylation age acceleration in girls but not boys in ALSPAC (Tang et al., 2020) – the study also used in this work. Moreover, the specific ACEs were found to be important – girls reporting emotional abuse or physical abuse showed the greatest acceleration in DNA methylation. Few studies have explored gender differences in associations between ACEs and early life inflammation to date, but there is suggestion that potential gender differences in associations begin to emerge in early life (Heard-Garris et al., 2020; Koss and Gunnar, 2017), although research to date has typically been on small, unrepresentative samples.

### 1.2 Aims of the present study

The aim of this study was to identify which specific ACEs or ACE clusters were related more strongly with inflammation in early life and whether associations differed for girls and boys. We hypothesised that ACEs involving physical harm or perceived threat of harm or abuse (e.g. physical abuse, sexual abuse, inter-parental violence) would be associated with higher levels of inflammation, following the work of Sheridan and McLaughlin (2014). We also hypothesised that associations between ACEs and inflammation would be stronger for girls compared to boys.

## 2 Methods

### 2.1 Dataset

In this study we used data from the Avon Longitudinal of Parents and Children (ALSPAC). ALSPAC is a prospective pre-natal cohort from the Avon region of South-West England. Pregnant women with expected delivery dates between 1st April 1991 to 31st December 1992 (n = 14,541, 71.8 % of eligible pregnancies) were included in the study (Boyd et al., 2012; Fraser et al., 2013). At approximately 7 years of age the sample was boosted with children with eligible birth dates who were not part of the initial sample. The total sample size for analyses using any data collected after the age of seven is therefore 15,454 pregnancies, resulting in 15,589 foetuses. Of these, 14,901 were alive at 1 year of age. The present study uses information from surveys from birth through to 17 years of age. Written informed consent was obtained from the parents of the cohort children. Study members have the right to withdraw their consent at any time. Further information on the ALSPAC consent procedures can be found here: http://www.bristol.ac.uk/alspac/researchers/research-ethics/

### 2.2 Measures

#### 2.2.1 Inflammation

Two inflammatory markers were used in this study – C-Reactive Protein (CRP) and Interleukin-6. At age 9 non-fasting blood samples were taken from the ALSPAC children enabling the analysis of both IL-6 and CRP. The blood samples were immediately spun, frozen and stored at -80℃. The automated particle-enhanced Tina-quant immunoturbidimetric assay (Roche UK) was used to measure high-sensitivity CRP. An enzyme-linked immunosorbent assay (ELISA) was used to capture IL-6 (Hayes et al., 2017). The intra and inter-assay values were less than 5% for both IL-6 and CRP. The minimum detection level for IL-6 was 0.1 pg/mL and 0.03 mg/L for CRP. IL-6 was observed for 4935 children and 4886 children had CRP values ≤10 mg/L (n = 49 had values >10 mg/L), representing the analytic samples for associations between ACEs and inflammation at age 9. Both inflammatory markers were log-transformed for subsequent analyses due to positive skew.

#### 2.2.2 Adverse childhood experiences (ACEs)

Ten ACEs between birth and age 8 years were included in the present study. We decided to focus on intra-familial adversities likely to require significant adaptation by the developing child (Lacey and Minnis, 2020). These ACEs included parental separation/divorce, parental alcohol problems, parental drug use, mother’s and father’s mental health problems, parental offending, inter-parental violence, physical abuse, emotional abuse, and sexual abuse. These experiences were reported by either the cohort child’s mother or the mother’s partner at 8 months, 1 year 9 months, 2 years 9 months, 3 years 11 months, 5 yrs 1 month, 6 year 1 month and 8 year 1 month.

Parental separation/divorce was reported by whether the mother had been separated or divorced from her partner since the previous survey. Parental alcohol misuse was defined as consuming ≥4 units of alcohol every day or self-reported alcoholism. Parental drug use was indicated by daily cannabis use or any use of hard drugs, such as amphetamines, heroin, cocaine, methadone, ecstasy or barbiturates. Mother’s and father’s mental health problems were indicated by a score of 13 or more on the Edinburgh Postnatal Depression Scale (EPDS) indicative of ‘probable depression’ (Boyd et al., 2005), self-reported suicide attempt or self- or partner-reported doctor consultations for schizophrenia, depression or anxiety. Parental convictions were self-reported by the mother and mother’s partner. Inter-parental violence was reported by the mother and mother’s partner; the respondent was asked whether their partner had physically hurt or been physically cruel to them. Child-directed physical abuse or ‘emotional cruelty’ by the mother or mother’s partner were coded as ‘Physical abuse’ or ‘Emotional abuse’, respectively. Sexual abuse was reported by the cohort child’s mother as ‘whether the child had been sexually abused by someone since xx’. Further information on these ACEs and the surveys in which they were measured are detailed in Appendix A.

#### 2.2.3 Covariates

Gender was included as a potential effect modifier in our analyses. Other covariates included *a priori* confounders which were also associated with both our ACE measures and inflammatory markers. These were whether the mother had smoked during pregnancy, the mother’s marital status at the time of birth of the cohort child (single, married or divorced/separated/widowed), the mother’s highest educational qualification by time of birth of the cohort child (categorised as Certificate of Secondary Education (CSE) or Ordinary (O)-Level, vocational qualification, Advanced (A)-Level, or degree or higher qualification), household crowding index (number of persons per room, categorised as ‘crowded’ if >1 persons per room), and the mother’s EPDS score during pregnancy. Please note that the study website contains details of all the data that is available through a fully searchable data dictionary and variable search tool http://www.bristol.ac.uk/alspac/researchers/our-data/

### 2.3 Multiple imputation

As missing information is a key source of bias, particularly in longitudinal studies (Sterne et al., 2009), we accounted for missing data using multiple imputation by chained equations. The imputation models included all analysis variables plus variables likely to be predictive of missingness, such as socioeconomic factors, ethnicity, and indicators of health. We imputed missing information for all cohort members with either IL-6 or CRP observed at age 9 (n = 4935) and restricted the subsequent analyses to those who had each outcome observed, following the Multiple Imputation then Deletion approach (Von Hippel, 2007). Twenty imputed datasets were created, and the distribution of observed and imputed data was similar (Table 1). For transparency, we also provide the findings from the complete case analysis in Appendix B.

**Table 1:** Descriptive characteristics of the study sample and comparison of observed and imputed data.

				Imputed					
	Observed			Overall		Boys		Girls	
	N/Median	%/[IQR]	Number missing	Median	%/ [IQR]	Median	%^a^/[IQR]	Median	%^a^/[IQR]
*Inflammatory markers - 9 years*									
IL-6, median [IQR]	0.81	[0.49, 1.42]	0	0.81	[0.49, 1.42]	0.71	[0.44, 1.23]	0.92	[0.56, 1.55]
CRP, median [IQR]	0.21	[0.11, 0.52]	0	0.21	[0.11, 0.54]	0.16	[0.10, 0.38]	0.28	[0.14, 0.69]
*ACEs 0−8 years*									
Parental separation	514	15.3	1583		11.1		10.7		11.6
Parental alcohol problems	138	10.5	3625		3.0		3.0		3.0
Mother's mental health problems	1055	39.0	2230		32.3		30.4		34.2
Father's mental health problems	238	20.1	3748		13.4		13.7		13.0
Parental convictions	86	5.5	3373		1.7		1.8		1.7
Inter-parental violence	132	8.9	3455		4.7		4.5		4.8
Physical abuse	516	18.1	2081		11.2		13.2		9.1
Emotional abuse	490	14.6	1575		10.1		10.6		9.7
Parental drug use	109	6.3	3214		2.3		2.5		2.2
Sexual abuse	14	0.4	1737		0.3		0.2		0.4
ACE score									
0 ACEs	200	34.5	4355^b^		41.2		41.9		40.5
1 ACE	175	30.2			30.4		30.0		30.8
2 ACEs	97	16.7			12.6		12.7		12.5
3 ACEs	61	10.5			5.1		4.7		5.5
4+ ACEs	47	8.1			10.7		10.7		10.7
*Covariates*									
Sex									
Boys	2490	50.5	0		50.5				
Girls	2445	49.5			49.5				
Mother smoked during pregnancy									
No	3730	85.9	593		87.3		87.5		87.1
Yes	612	14.1			12.7		12.5		12.9
Mother's marital status									
Single	501	11.4	536		10.7		10.0		11.3
Widowed, divorced/separated	194	4.4			4.0		3.7		4.2
Married	3704	84.2			85.4		86.3		84.5
Mother's highest educational qualification									
CSE or O level	2178	48.1	400		50.5		50.3		50.7
Vocational	353	7.8			7.2		7.5		6.8
A level	1229	27.1			26.4		26.2		26.7
Degree	775	17.1			16.0		16.1		15.8
Overcrowding									
≤1 persons per room	4311	95.9	441		96.3		96.2		96.3
>1 persons per room	183	4.1			3.8		3.8		3.7
Mother's EPDS score during pregnancy, median [IQR]	6 [3, 9]		559		6 [3, 9]		6 [3, 9]		6 [4, 9]

Descriptive characteristics are reported for those who have CRP or IL-6 observed at age 9 (n = 4935).

a Only pooled percentages are presented for the imputed data as the specific Ns will vary across imputed datasets.

b There is a high level of missing data on ACE score but the ACE score was actually computed post-imputation and so the version of the variable shown here is purely for demonstration purposes. Abbreviations: ACEs = adverse childhood experiences; CRP = C-Reactive Protein; CSE = Certificate of Secondary Education; EPDS = Edinburgh Postnatal Depression Score; IL-6 = Interleukin-6; IQR = Interquartile range.

### 2.4 Operationalising ACEs

In this study we compared three ways of operationalising ACEs information. First we considered each ACE individually. Second, we looked at cumulative risk whereby ‘ACE scores’ were created after imputing missing values on the individual ACE items by adding together the number of ACEs experienced. Consistent with previous research this was categorised as 0 ACEs, 1 ACE, 2 ACEs, 3 ACEs or 4+ ACEs (Felitti et al., 1998). Thirdly, we applied LCA using the robust maximum likelihood estimator to our ACEs data to identify distinct clusters of individuals co-reporting similar ACEs. We compared models for 2–6 classes. The best fitting class solution was determined by comparing the model fit indices – Akaike’s Information Criteria (AIC), Bayesian Information Criteria (BIC) and the sample-size adjusted Bayesian Information Criteria (SSABIC). Lower values of the AIC, BIC and SSABIC indicate a better fitting model. Also an entropy value approaching 1 suggests better distinction of classes. In deciding on the best fitting model we gave preference to improvements in the BIC, as recommended (Nylund-Gibson and Young Choi, 2018). Upon selecting the optimal class solution, cohort members were assigned to their most likely class, thereby creating a categorical variable for use in subsequent analyses. A latent profile plot was constructed for the final class solution to aid with the qualitative interpretation of the derived classes.

### 2.5 Testing associations between ACEs and inflammation

We tested associations between the three methods of ACEs operationalisation with IL-6 and CRP at age 9 using linear regression. Two sets of models were run - the first was the crude association between each ACE operationalisation and inflammation; the second included all covariates. In interaction term between each ACE operationalisation variable and gender was tested in the covariate-adjusted models. The results of all regression models are expressed as percentage difference to aid interpretation (Cole and Altman, 2017) and raw logIL-6 and logCRP coefficients provided in appendices. All of the data cleaning, multiple imputation and regression was conducted in Stata version 15.1. The latent class analysis was conducted in MPlus version 7.3.

## 3 Results

The characteristics of the study sample are presented in Table 1. Median values of inflammatory markers were low at age 9.. Levels of inflammation were higher for girls than boys on both measures. The most commonly reported ACE was maternal mental health problems (32.3 %), followed by paternal mental health problems (13.4 %), physical abuse (11.2 %), parental separation/divorce (11.1 %) and emotional abuse (10.1 %). The least commonly reported ACE was sexual abuse (0.3 %). More boys reported physical abuse than girls (13.2 % versus 9.1 %) but there was little difference in the reporting of other ACEs by gender. More than half (58.8 %) of the sample reported at least one ACE, 30.4 % reported one ACE and more than 10 % reported four or more ACEs.

### 3.1 Deriving LCA-derived ACE classes

**Fig. 1:**
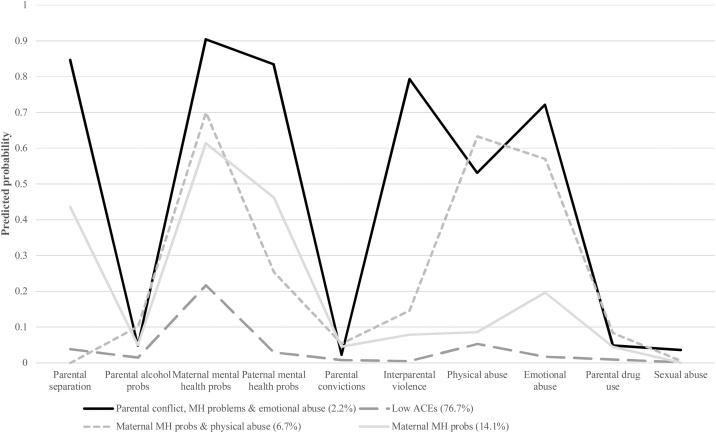
Profile plot for the four class solution.

The four class solution was found to be the best fitting model according to the BIC. The model fit of the 2–6 class models is shown in Appendix C and the average probability of class assignment for each class shown in Appendix D. Table 2 shows the predicted probabilities of each ACE in each of the four classes and the corresponding profile plot is shown in Fig. 1. The largest class (76.7 % of cohort members) was the ‘Low ACEs’ class, comprised of cohort members with low probability of reporting any ACEs. The second largest class comprised of individuals likely to report ‘Maternal mental health problems’ only (14.1 %). The third class was comprised of individuals likely to report ‘Maternal mental health problems and physical abuse’ (6.7 %). Finally, the smallest group represented cohort members reporting parental separation/divorce, inter-parental violence, maternal and paternal mental health problems, and emotional abuse (‘Parental conflict, mental health problems and emotional abuse’, 2.2 %).

**Table 2:** Predicted probabilities of ACEs in each latent class in the four class solution.

	Low ACEs (76.7 %)	Maternal mental health problems (14.1 %)	Maternal mental health problems & physical abuse (6.7 %)	Parental conflict, mental health problems & emotional abuse (2.2 %)
Parental separation/divorce	0.04	0.44	0.003	0.85
Parental alcohol problems	0.01	0.05	0.10	0.05
Maternal mental health problems	0.22	0.61	0.70	0.90
Paternal mental health problems	0.03	0.46	0.25	0.83
Parental convictions	0.01	0.05	0.05	0.02
Inter-parental violence	0.005	0.08	0.15	0.79
Physical abuse	0.05	0.09	0.63	0.53
Emotional abuse	0.02	0.20	0.57	0.72
Parental drug use	0.01	0.04	0.08	0.05
Sexual abuse	0.002	0.003	0.01	0.04

### 3.2 Associations between ACEs and inflammation

The results of the linear regression analysis testing associations between the three ACE operationalisations and inflammation are shown in Table 3 (presented as % difference) and in Appendix E as logCRP and logIL-6. The number of ACEs reported by age 8 was not associated with either IL-6 or CRP at age 9. Looking at individual ACEs, parental separation/divorce was associated with higher IL-6 in the covariate-adjusted model (14.31 % higher, 95 % CI: 5.44, 23.92). In contrast, parental alcohol problems, paternal mental health problems, parental convictions and emotional abuse were associated with lower levels of IL-6 at age 9. No associations were seen between individual ACEs and CRP at age 9. There was also no evidence of associations between LCA-derived ACE clusters and inflammation.

**Table 3:** Associations between ACEs operationalisations and inflammation at age 9.

	IL-6 (n = 4935)				CRP (n = 4887)			
	Crude model		Covariate-adjusted^a^		Crude model		Covariate-adjusted^a^	
	% difference	95 % CI	% difference	95 % CI	% difference	95 % CI	% difference	95 % CI
ACE score								
0 ACEs	Ref		Ref		Ref		Ref	
1 ACE	3.28	−2.53, 9.43	2.31	−3.55, 8.51	1.66	−5.78, 9.68	1.09	−6.44, 9.21
2 ACEs	0.68	−6.88, 8.85	−0.92	−8.54, 7.32	−7.05	−16.13, 3.01	−7.80	−17.03, 2.45
3 ACEs	5.51	−5.80, 18.18	3.56	−7.73, 16.24	−0.31	−14.30, 15.97	−0.92	−15.08, 15.60
4+ ACEs	8.33	−0.30, 17.71	3.83	−5.16, 13.68	−6.80	−16.46, 3.97	−8.41	−18.73, 3.21
Individual ACEs								
Parental separation/divorce	17.47	8.78, 26.85	14.31	5.44, 23.92	2.30	−7.57, 13.21	2.32	−8.03, 13.83
Parental alcohol problems	−13.57	−25.04, -0.34	−13.56	−25.05, -0.31	−6.64	−22.50, 12.47	−5.83	−21.86, 13.50
Maternal mental health problems	7.11	1.71, 12.80	5.59	−0.41, 11.95	−0.45	−7.02, 6.58	−0.91	−8.27, 7.05
Paternal mental health problems	−4.99	−11.52, 2.02	−7.41	−13.93, -0.40	−6.58	−14.96, 2.62	−6.63	−15.22, 2.82
Parental convictions	−20.35	−33.80, -4.16	−19.80	−33.35, -3.49	−8.55	−28.30, 16.65	−7.99	−27.91, 17.42
Inter-parental violence	9.61	−2.28, 22.95	6.83	−4.92, 20.04	0.49	−13.53, 16.78	0.73	−13.54, 17.36
Physical abuse	−0.73	−8.08, 7.20	−0.63	−8.05, 7.40	−0.60	−10.14, 9.95	0.39	−9.36, 11.19
Emotional abuse	−8.48	−15.54, -0.83	−9.36	−16.42, -1.70	−10.16	−19.15, -0.18	−9.75	−18.89, 0.41
Parental drug use	−3.78	−18.11, 13.05	−4.23	−18.51, 12.55	−17.77	−33.76, 2.08	−17.17	−33.32, 2.90
Sexual abuse	16.41	−26.17, 83.55	13.48	−28.02, 78.91	22.88	−32.23, 122.83	22.91	−32.27, 123.06
LCA-derived ACE clusters								
Low ACEs (81.1 %)	Ref		Ref		Ref		Ref	
Maternal mental health problems (10.3 %)	3.59	−4.10, 12.25	0.85	−7.11, 9.49	−8.23	−17.48, 2.06	−8.95	−18.35, 1.53
Maternal mental problems + physical abuse (6.3 %)	−6.03	−14.99, 3.87	−6.48	−15.50, 3.50	−5.12	−16.80, 8.19	−4.68	−16.58, 8.90
Parental conflict, mental health problems & emotional abuse (2.4 %)	15.20	−1.85, 35.22	8.03	−8.43, 27.45	4.98	−14.83, 29.44	4.09	−16.18, 29.26

a Adjusted for overcrowding, maternal smoking during pregnancy, maternal EPDS score during pregnancy, mother’s marital status & mother’s highest educational qualification; Abbreviations: ACEs = adverse childhood experiences; CI = Confidence Interval; CRP = C-Reactive Protein; IL-6 = Interleukin-6.

### 3.3 Gender differences in associations between ACEs and inflammation

No gender differences were observed in associations between ACE score and either inflammatory marker. However, when we explored associations between individual ACEs and inflammation, boys with a father with mental health problems had lower levels of IL-6 which were not observed in girls (Table 4 as % difference, Appendix F as logIL-6). Similarly, boys who experienced emotional abuse had lower levels of IL-6 (15.85 % lower, 95 % CI: -24.95, -5.65) but girls experiencing emotional abuse did not. In contrast there was suggestion that the relationship between parental drug use and IL-6 was stronger for girls than boys. None of the associations between individual ACEs and CRP differed for boys and girls. However, for the LCA-derived clusters, ‘Maternal mental health problems’ demonstrated a stronger relationship with CRP for boys than girls.

**Table 4:** Gender differences in associations between ACEs and inflammation.

	IL-6 (n = 4935)				CRP (n = 4887)			
	Boys		Girls		Boys		Girls	
	logIL-6	95 % CI	logIL-6	95 % CI	logCRP	95 % CI	logCRP	95 % CI
Individual ACEs								
Paternal mental health problems	−14.55	−22.92, -5.27	1.92	−7.93, 12.82				
Emotional abuse	−15.85	−24.95, -5.65	0.03	−10.71, 12.06				
Parental drug use	12.52	−10.13, 40.88	−19.88	−36.28, 0.74				
LCA-derived clusters								
Maternal mental health problems					−20.60	−31.58, -7.86	4.76	−10.12, 22.11

NB models shown are adjusted for overcrowding, maternal smoking during pregnancy, maternal EPDS score during pregnancy, mother’s marital status & mother’s highest educational qualification; Abbreviations: ACEs = adverse childhood experiences; CI = Confidence Interval; CRP = C-Reactive Protein; IL-6 = Interleukin-6.

## 4 Discussion

### 4.1 Summary of findings

We explored whether specific ACEs or ACE combinations were related to inflammatory markers at age 9. The ACE score was not associated with inflammatory markers at age 9. Parental separation/divorce was associated with higher IL-6, and parental alcohol problems, paternal mental health problems, parental convictions and emotional abuse were all associated with lower levels of IL-6. The associations for paternal mental health problems and emotional abuse were only observed for boys. None of the individual ACEs were associated with CRP at age 9. ACEs were found to cluster in this cohort. The ‘Maternal mental health problems’ cluster was associated with lower CRP for girls in this sample. No other associations were observed for CRP..

### 4.2 Interpretation of findings

Recent research using ALSPAC found that CRP at age 9 did not mediate associations between a total score of prenatal and childhood stressors and adolescent depressive symptoms (Flouri et al., 2020). But they found that an increase in the number of stressors over early life was associated with higher IL-6 at age 9. However the number of stresses included was very diverse and extensive. The authors did not investigate associations between individual ACEs and inflammation, and our results suggest that these associations may be driven by specific ACEs; in our study parental separation/divorce was associated with higher levels of IL-6 at age 9. Slopen et al. (2012) showed that an ACE score and specific ACEs were associated with higher levels of IL-6 and CRP at age 9 in ALSPAC, particularly when those ACEs had been experienced recently (i.e. at age 7 or 8). Whilst this is not consistent with our findings, the ACEs that were investigated in that study differed from ours; Slopen et al. (2012) focused on five ACEs – whether the child was taken into foster care, sexual abuse, whether the child was physically hurt by someone, and whether the child had been separated from their mother or father. This discrepancy in study findings further points to the importance of individual ACEs in producing differences in inflammatory responses, potentially produced via different physiological mechanisms.

Contrary to our expectations we observed that boys had lower levels of IL-6 if they had experienced paternal mental health problems or emotional abuse, but these associations were not observed in girls. In contrast there was some suggestion that girls but not boys who had experienced parental drug use had lower levels of IL-6. Previous studies examining associations between early life adversities and inflammation in ALSPAC have not previously tested gender interactions, and our findings suggest that associations between early life adversities and inflammation vary by gender. Further, in this study we found that some ACEs - parental alcohol problems, paternal mental health problems (for boys), parental convictions and emotional abuse (for boys) – were associated with lower levels of IL-6 at age 9. This was contrary to our expectations, particularly as much of the psychoneuroendocrinology literature focuses on chronic, low-grade inflammation as a key pathway between social stressors, such as ACEs, and health outcomes. However, this assumption is becoming increasingly questioned on the basis of recent immunological research (Steel et al., 2020). IL-6 has two signalling pathways – one involved in the inflammatory response and the other role being invoked in the absence of inflammation. Consequently, IL-6 might have an anti- rather than pro-inflammatory role (Del Giudice and Gangestad, 2018), and so we cannot assume that social stressors will always result in an *increase* in inflammatory markers as the physiological processes involved are far more complex. It is perhaps becoming clear that we do not fully understand what differences in IL-6 mean, particularly in early life, what implications these differences have for later health. Indeed, in the present study we find that four ACEs were related to lower than average IL-6 levels and only one ACE (parental separation/divorce) was associated with higher levels of IL-6. Whilst this is consistent with IL-6′s potential anti-inflammatory role, further research is needed from physiological studies as to how these biological processes might operate and in response to which types of stressor. Consequently, the present study not only highlights the issues of measurement of ACEs but also of measurement of inflammatory markers in early life.

Whilst previous work has shown that individual ACEs (Lacey et al., 2014, 2013), ACE scores (Chen and Lacey, 2018) and LCA-derived clusters (R. E. Lacey et al., 2020b) were associated with higher levels of inflammatory markers in mid-life in the NCDS, it appears from the present findings that few differences are evident by age 9 and that differences in inflammatory responses begin to emerge later in the life course. Indeed a meta-analysis of 9 studies investigating relationships between early life adversity and CRP and IL-6 presented evidence of a very small effect (Kuhlman et al., 2020). There was an interesting interaction by age, with the largest effects being evident in infancy and adolescence, but few differences observed in between. However, the analysis of age interactions was under-powered as few studies have investigated associations between ACEs in middle to late childhood, particularly utilising large population studies. This is one aspect to which the present study adds. Kuhlman et al. (2020) also identified evidence of publication bias in studies investigating associations between ACEs and IL-6, suggesting that null (or even perhaps negative) effects pertaining to IL-6 might be under-published.

Given that differences in inflammatory markers in response to ACEs might emerge later in life, this is helpful when thinking about the mechanisms involved. One of the key mechanisms linking ACEs to inflammation involves health damaging behaviours, which very few young people will be engaged with by later childhood (Kelly et al., 2016). Differences in health damaging behaviours in response to ACEs appear to emerge later in adolescence (Kisely et al., 2019), and this may be one reason why we saw few associations between ACEs and inflammation at an earlier age. Further research which utilises repeated measures of ACEs, inflammation and mediators (e.g. health behaviours, mental health) is needed to unpick when differences in inflammatory responses emerge and via what mechanisms.

### 4.3 Strengths and limitations

This study is not without its limitations. As mentioned above, we investigated relationships between ACEs and IL-6 or CRP – inflammatory markers that are frequently used in studies on ACEs and inflammation. IL-6 and CRP are frequently used in psychoneuroendocrinology studies investigating associations with the early life environment, but mainly in adults. Recently there was a call for more studies which investigate different inflammatory markers, such as TNF-α (Kuhlman et al., 2020). Recent work also suggests that soluble urokinase plasminogen activator receptor (suPAR), a novel inflammatory marker indicating the overall level of immune functioning, is associated with ACEs in individuals where CRP is not (Rasmussen et al., 2020). Similarly, GlycA is emerging as a promising marker of systemic inflammation in population studies (Connelly et al., 2017). Future research should therefore investigate whether ACEs are related to these markers of inflammation across the life course, but particularly in early life. Whilst being a useful method for identifying ACE clusters, LCA is data- rather than theoretically-driven. The findings might also be dataset-specific, although with the increasing application of this method to multiple datasets some similarities in LCA-derived clusters are emerging.

This study has many strengths. First, we used a large longitudinal population sample from the UK to explore the relationships between ACEs and inflammation in. early life – one of the largest studies of its kind. Second, given that much of the research into ACEs and biological sequelae has been conducted on adult populations, there has been a reliance on retrospective ACE reports (Danese, 2018). There is now greater recognition that there is poor agreement between retrospective and prospective measures of ACEs e.g.(Baldwin et al., 2019). As such, these two reporting methods cannot be assumed to be interchangeable. Further, children who have prospective ACEs information might have different pathways to psychopathology than adults retrospectively reporting ACEs information (Baldwin et al., 2019), and these cannot be assumed to be the same. Few studies thus far have investigated association between prospectively collected ACEs information and inflammation in early life, a key strength of the present study. Third, missing data is a key source of bias in longitudinal population studies. In the present study we attempted to account for bias arising from missing data by implementing multiple imputation. The results of the complete case analysis shown in Appendix B suggest that the study is underpowered without applying this approach, although the findings and conclusions are largely similar to those using the imputed data. Finally, by applying a LCA approach we explored person-centred clustering of ACEs and their relationships with ACEs. We were able to derive four clusters representing the way in which ACEs cluster in this sample, although here they were not associated with IL-6 or CRP.

## 5 Conclusions

In summary, we showed that ACEs cluster in the ALSPAC cohort and derived four clusters using an LCA approach. However, these ACE clusters were not associated with early life inflammation and neither were ACE scores. However, specific ACEs were associated with IL-6 at age 9 suggesting that parental separation/divorce was associated with higher levels of IL-6 but that other ACEs (parental alcohol problems, paternal mental health problems, parental convictions and emotional abuse) were associated with lower levels of IL-6. Therefore the examination of specific ACEs is likely to be very important in continued investigations of the link between adversity and inflammation, and in summarising ACEs - either through clustering or ACE scores - might miss important relationships and mechanisms. Further research is needed to tease out when inflammatory responses to ACEs emerge, perhaps using repeated measures of ACEs and novel markers of inflammation and immune functioning. We also need more research into the importance of raised or lowered inflammation in early life, and what consequences this might have longer-term for ongoing health and disease risk.

## Funding

This work was supported by the Wellcome Trust ALSPAC core programme grant [102215]. RL is supported by the UK Economic and Social Research Council (ESRC) (grant number ES/P010229/1). EI is funded by the ESRC-BBSRC Soc-B Centre for Doctoral Training (ES/P000347/1). LDH is supported by a Career Development Award from the UK Medical research Council (MR/M020894/1). The UK Medical Research Council and Wellcome (grant ref: 217065/Z/19/Z) and the University of Bristol provide core support for ALSPAC. This publication is the work of the authors and RL will serve as guarantor for the contents of this paper. A comprehensive list of grants funding is available on the ALSPAC website (http://www.bristol.ac.uk/alspac/external/documents/grant-acknowledgements.pdf); this research was specifically funded by the National Institute of Health (R01 DK077659). The funders played no role in the study design, collection of data and analysis, the interpretation of results, decision to publish, or the preparation of the manuscript.

## Declaration of Competing Interest

The authors reported no declarations of interest.

## Figures and Tables

**Table:** 




**Table:** 

	IL-6 (n = 567)				CRP (n = 562)			
	Crude model		Covariate-adjusted^a^		Crude model		Covariate-adjusted^a^	
	logIL6	95 % CI	logIL6	95 % CI	logCRP	95 % CI	logCRP	95 % CI
ACE score								
0 ACEs	Ref		Ref		Ref		Ref	
1 ACE	0.03	−0.16, 0.21	0.01	−0.18, 0.20	0.07	−0.15, 0.30	0.08	−0.15 0.31
2 ACEs	−0.01	−0.23, 0.21	−0.03	−0.26, 0.20	0.02	−0.26, 0.29	0.04	−0.24, 0.32
3 ACEs	0.03	−0.22, 0.29	0.02	−0.24, 0.29	0.05	−0.27, 0.37	0.05	−0.28, 0.38
4+ ACEs	0.03	−0.26, 0.32	−0.03	−0.32, 0.27	0.15	−0.21, 0.50	0.12	−0.24, 0.48
Individual ACEs								
Parental separation/divorce	0.23	−0.09, 0.54	0.26	−0.08, 0.59	0.17	−0.21, 0.56	0.22	−0.19, 0.64
Parental alcohol problems	−0.01	−0.25, 0.23	−0.06	−0.31, 0.20	0.001	−0.29, 0.30	−0.01	−0.32, 0.30
Maternal mental health problems	0.01	−0.14, 0.17	−0.01	−0.17, 0.15	0.02	−0.17, 0.21	0.04	−0.15, 0.23
Paternal mental health problems	0.01	−0.18, 0.19	−0.03	−0.22, 0.16	−0.04	−0.26, 0.18	−0.06	−0.29, 0.17
Parental convictions	−0.001	−0.32, 0.32	0.01	−0.31, 0.33	0.11	−0.28, 0.49	0.14	−0.25, 0.53
Inter-parental violence	0.09	−0.16, 0.34	0.07	−0.18, 0.33	0.14	−0.17, 0.44	0.10	−0.21, 0.41
Physical abuse	−0.001	−0.18, 0.18	0.004	−0.18, 0.19	0.02	−0.20, 0.24	0.04	−0.18, 0.26
Emotional abuse	−0.21	−0.41, -0.01	−0.23	−0.43, -0.02	0.07	−0.18, 0.32	0.02	−0.24, 0.27
Parental drug use	0.09	−0.23, 0.41	0.005	−0.33, 0.34	−0.07	−0.47, 0.34	−0.15	−0.57, 0.27
Sexual abuse	1.07	−0.69, 2.84	1.10	−0.66, 2.86	1.99	−0.16, 4.14	1.99	−0.17, 4.14
LCA-derived ACE clusters								
Low ACEs (81.1 %)	Ref		Ref		Ref		Ref	
Maternal mental health problems (10.3 %)	3.59	−4.10, 12.25	0.85	−7.11, 9.49	−8.23	−17.48, 2.06	−8.95	−18.35, 1.53
Maternal mental problems + physical abuse (6.3 %)	−6.03	−14.99, 3.87	−6.48	−15.50, 3.50	−5.12	−16.80, 8.19	−4.68	−16.58, 8.90
Parental conflict, mental health problems & emotional abuse (2.4 %)	15.20	−1.85, 35.22	8.03	−8.43, 27.45	4.98	−14.83, 29.44	4.09	−16.18, 29.26

**Table:** 

	Number of classes				
	2	3	4	5	6
BIC	30403.8	30290.2	**30244.934**	30277.3	30327.9
AIC	30261.5	30073.5	**29953.7**	29911.5	29887.6
SSABIC	30,337	30188.5	**30108.3**	30105.7	30121.3
entropy	0.76	0.84	**0.76**	0.71	0.73

**Table:** 

		Assigned latent class			
		‘Parental conflict, mental health problems & emotional abuse’	‘Low ACEs’	‘Maternal mental health problems & physical abuse’	‘Maternal mental health problems’
Most likely latent class membership	‘Parental conflict, mental health problems & emotional abuse’	0.839	0.000	0.046	0.114
	‘Low ACEs’	0.000	0.915	0.023	0.062
	‘Maternal mental health problems & physical abuse’	0.026	0.100	0.693	0.182
	‘Maternal mental health problems’	0.048	0.144	0.054	0.753

**Table:** 

	IL-6 (n = 4935)				CRP (n = 4887)			
	Crude model		Covariate-adjusted^a^		Crude model		Covariate-adjusted^a^	
	logIL-6	95 % CI	logIL-6	95 % CI	logCRP	95 % CI	logCRP	95 % CI
ACE score								
0 ACEs	Ref		Ref		Ref		Ref	
1 ACE	0.03	−0.03, 0.09	0.02	−0.04, 0.08	0.02	−0.06, 0.92	0.01	−0.07, 0.09
2 ACEs	0.01	−0.07, 0.08	−0.01	−0.09, 0.07	−0.07	−0.18, 0.03	−0.08	−0.19, 0.02
3 ACEs	0.05	−0.06, 0.17	0.04	−0.08, 0.15	−0.003	−0.15, 0.15	−0.01	−0.16, 0.14
4+ ACEs	0.08	−0.003, 0.16	0.04	−0.05, 0.13	−0.07	−0.18, 0.04	−0.09	−0.21, 0.03
Individual ACEs								
Parental separation/divorce	0.16	0.08, 0.24	0.13	0.05, 0.21	0.02	−0.08, 0.12	0.02	−0.08, 0.13
Parental alcohol problems	−0.15	−0.29, -0.003	−0.15	−0.29, -0.003	−0.07	−0.25, 0.12	−0.06	−0.25, 0.13
Maternal mental health problems	0.07	0.02, 0.12	0.05	−0.004, 0.11	−0.005	−0.07, 0.06	−0.01	−0.09, 0.07
Paternal mental health problems	−0.05	−0.12, 0.02	−0.08	−0.15, -0.004	−0.07	−0.16, 0.03	−0.07	−0.17, 0.03
Parental convictions	−0.23	−0.41, -0.04	−0.22	−0.41, -0.04	−0.09	−0.33, 0.15	−0.08	−0.33, 0.16
Inter-parental violence	0.09	−0.02, 0.21	0.07	−0.05, 0.18	0.005	−0.15, 0.16	0.01	−0.15, 0.16
Physical abuse	−0.01	a−0.08, 0.07	−0.01	−0.08, 0.07	−0.01	−0.11, 0.09	0.004	−0.10, 0.11
Emotional abuse	−0.09	−0.17, -0.01	−0.10	−0.18, -0.02	−0.11	−0.21, -0.002	−0.11	−0.21, 0.004
Parental drug use	−0.04	−0.20, 0.12	−0.04	−0.20, 0.12	−0.20	−0.41, 0.02	−0.19	−0.41, 0.03
Sexual abuse	0.15	−0.30, 0.61	0.13	−0.33, 0.58	0.21	−0.39, 0.80	0.21	−0.39, 0.80
LCA-derived ACE clusters								
Low ACEs (81.1 %)	Ref		Ref		Ref		Ref	
Maternal mental health problems (10.3 %)	0.04	−0.04, 0.12	0.01	−0.07, 0.09	−0.09	−0.19, 0.02	−0.09	−0.20, 0.02
Maternal mental problems + physical abuse (6.3 %)	−0.06	−0.16, 0.04	−0.07	−0.17, 0.03	−0.05	−0.18, 0.08	−0.05	−0.18, 0.09
Parental conflict, mental health problems & emotional abuse (2.4 %)	0.14	−0.02, 0.30	0.08	−0.09, 0.24	0.05	−0.16, 0.26	0.04	−0.18, 0.26

**Table:** 

	IL-6 (n = 4935)				CRP (n = 4887)			
	Boys		Girls		Boys		Girls	
	logIL-6	95 % CI	logIL-6	95 % CI	logCRP	95 % CI	logCRP	95 % CI
Individual ACEs								
Paternal mental health problems	−0.16	−0.26, -0.05	0.02	−0.08, 0.12				
Emotional abuse	−0.17	−0.29, -0.06	0.0003	−0.11, 0.11				
Parental drug use	0.12	−0.11, 0.34	−0.22	−0.45, 0.01				
LCA-derived clusters								
Maternal mental health problems					−0.23	−0.38, -0.08	0.05	−0.11, 0.20

## Appendix B Results from complete case analyses

^a^Adjusted for overcrowding, maternal smoking during pregnancy, maternal EPDS score during pregnancy, mother’s marital status & mother’s highest educational qualification; Abbreviations: ACEs = adverse childhood experiences; CI = Confidence Interval; CRP = C-Reactive Protein; IL-6 = Interleukin-6

## Appendix C Model fit indices for the 2–6 class latent class solutions

Abbreviations: AIC Akaike information criterion; BIC Bayesian information criterion; SSABIC Sample size adjusted Bayesian information criterion

## Appendix E Associations between ACEs operationalisations and inflammation at age 9 (log output)

^a^Adjusted for overcrowding, maternal smoking during pregnancy, maternal EPDS score during pregnancy, mother’s marital status & mother’s highest educational qualification; Abbreviations: ACEs = adverse childhood experiences; CI = Confidence Interval; CRP = C-Reactive Protein; IL-6 = Interleukin-6

## Appendix F Gender differences in associations between ACEs and inflammation (log output)

NB models shown are adjusted for overcrowding, maternal smoking during pregnancy, maternal EPDS score during pregnancy, mother’s marital status & mother’s highest educational qualification; Abbreviations: ACEs = adverse childhood experiences; CI = Confidence Interval; CRP = C-Reactive Protein; IL-6 = Interleukin-6
